# Rab-mediated vesicle trafficking in cancer

**DOI:** 10.1186/s12929-016-0287-7

**Published:** 2016-10-06

**Authors:** Hong-Tai Tzeng, Yi-Ching Wang

**Affiliations:** 1 Department of Pharmacology, National Cheng Kung University, College of Medicine, No.1, University Road, Tainan, 70101 Taiwan People’s Republic of China; 2 Institute of Basic Medical Sciences, College of Medicine, National Cheng Kung University, No.1, University Road, Tainan, 70101 Taiwan People’s Republic of China

**Keywords:** Rab protein, Effector, Vesicle trafficking, Cancer

## Abstract

A large group of small Rab GTPases which mediate secretory and endosomal membrane transport, as well as autophagosome biogenesis, are essential components of vesicle trafficking machinery. Specific Rab protein together with the cognate effectors coordinates the dynamics of trafficking pathway and determines the cargo proteins destination. Functional impairments of Rab proteins by mutations or post-translational modifications disrupting the regulatory network of vesicle trafficking have been implicated in tumorigenesis. Therefore, the vesicle transport regulators play essential roles in the mediation of cancer cell biology, including uncontrolled cell growth, invasion and metastasis. The context-dependent role of the same Rab to act as either an oncoprotein or tumor suppressor in different cancers is found. Such discrepancies may be due in part to the interaction of specific Rab protein with different effectors or cargos in various tumors. Here, we review recent advances in the roles of Rab GTPases in communicating with other effectors in tumor progression. In this review, we also emphasize dysregulation of Rab-mediated membrane delivery shifting normal cell behaviors toward malignancy. Thus, recovery of the dysregulated vesicle trafficking systems in cancer cells may provide future directions for potential strategy to restrain tumor progression.

## Background

Rab proteins are evolutionarily conserved with 55–75 % identity across species. They are small GTPases comprising more than 70 members in humans and function as regulators of vesicles transport, proteins trafficking, membrane targeting and fusion [1–3]. Rab protein activity is controlled by cycling between the active GTP-bound and inactive GDP-bound forms. Guanine nucleotide exchange factors (GEFs) serve as the effectors of Rab GTPase by facilitating the exchange of GDP for GTP, resulting in the activation of Rabs and the downstream signaling [4]. In contrast, GTPase-activating proteins (GAPs) catalyze the hydrolysis of GTP to GDP to convert the GTP-bound Rabs to inactive GDP-bound form [5]. Some Rab small GTPases localize to the cytosol by forming complex with guanine dissociation inhibitors (GDIs) that prevent their membrane anchorage. Other effectors such as motor proteins, tethering factors and SNAREs (Soluble N-ethylmaleimide sensitive factor attachment protein receptor) are involved in the coordination of Rabs-mediated vesicle transport from donor membrane budding toward acceptor membrane fusion [6–8]. Dysregulation in Rabs level or mutations altering GTP/GDP-binding of Rabs or Rabs interaction with effectors may dampen the efficiency and specificity in membrane traffic that are implicated in disease development such as cancer.

In recent years, advanced progress in understanding the cellular functions of Rabs on vesicle trafficking has been made. Therefore, this review provides an overview of our current knowledge of regulation of Rab-mediated vesicle dynamics, and their critical roles in tumorigenesis.

### Rab GTPases function as molecular switches in membrane traffic

Over the past two decades, emerging evidence has shown that distinct classes of small GTPases are involved in membrane vesicles trafficking. Individual Rab protein localizes to the surface membrane of different organelle in the cytosolic compartment and regulates a specific membrane trafficking pathway for appropriate protein sorting and targeting. Most Rabs are expressed ubiquitously, while some have tissue/cell-type specificity. For example, Rab17 is predominantly expressed in epithelial cells and localized to apical recycling endosome (ARE) to mediate transcytosis to the basolateral plasma membrane [9]. Rab15 and Rab25 are also involved in the transportation of cargos through the ARE system [10, 11]. Rab12 is highly expressed in Sertoli cells and is responsible for the cargo delivery from peripheral to the perinuclear region to maintain centrosomes integrity [12]. Rab10 is expressed in adipocytes and implicated in mediating insulin-stimulated plasma membrane translocation of glucose transporter GLUT4 [13]. Rab8A and Rab13 are expressed in skeletal muscle cells and become active forms in response to insulin stimulation [14].

Some Rabs localize at different subcellular organelles. For instance, localization of Rab33 at the medial Golgi helps in the intra-Golgi transportation of vesicles [15]. Rab5 and Rab21 are involved in early endosome transport and mediate endocytosis pathway while Rab7 regulates cargo trafficking from early endosome to late endosome and subsequently to lysosome for degradation [16–18]. Another vesicle transport route is from trans-Golgi network (TGN) to the plasma membrane, which is mediated by secretory granules and vesicles. A lot of Rab proteins are associated with exocytic pathway including Rab3, Rab11, Rab26, Rab27, Rab37 and Rab38 [19–24]. Vesicles transport between TGN and early endosome are controlled by Rab22 and Rab31 [25, 26]. The diversity of individual Rab binding partner determines the specific vesicle transport route and creates the complexity of membrane trafficking.

Autophagy is responsible for degradation of intracellular components by transporting them to lysosomes to maintain cellular homeostasis and prevent pathogens infection. Rab proteins are also involved in the regulation of autophagy biogenesis [27]. Rab5, for example, participates in autophagy induction by the sequential signaling cascade in response to growth factor [28]. Overexpression of Rab32 promotes autophagosome biogenesis [29]. Rab33 has also been reported to regulate autophagosome formation through interaction with ATG16L, an essential factor for LC3 lipidation and membrane biogenesis in autophagy [30]. Several Rabs including Rab7, Rab11, Rab24 and Rab25 play critical roles in modulating autophagosome maturation [31–34]. In ovarian cancer cells, knockdown of Rab25 increases the conversion of LC3-I to LC3-II, a critical step for autophagy, and induces apoptosis. These results indicate a role of Rab25 in tumorigenesis relevant to autophagy suppression [34].

Emerging evidence has shown that exosomes act as a novel mode of intercellular communication. They deliver message from cancer cells to surrounding stromal cells as well as distant metastatic sites to create a pre-metastatic niche [35]. Exosome secretion is regulated by fusion of the plasma membrane with multivesicular bodies (MVBs), which are late endosomal structure of endocytic pathway containing intraluminal vesicles. It has been observed that Rab proteins critically contribute to exsosome release. For example, Rab11 regulates transferrin receptor secretion via the exosome pathway [36]. Similarly, Rab35 promotes exosome release by interacting with its effector TBC1D10A-C [37]. The role of Rabs in regulation of exosome pathway also has been explored in association with tumor progression. Indeed, Rab27-dependent exosome secretion of microRNAs is linked to tumor invasiveness in bladder cancer [38].

### Dysregulated Rab GTPases implicated in cancer

**Fig. 1:**
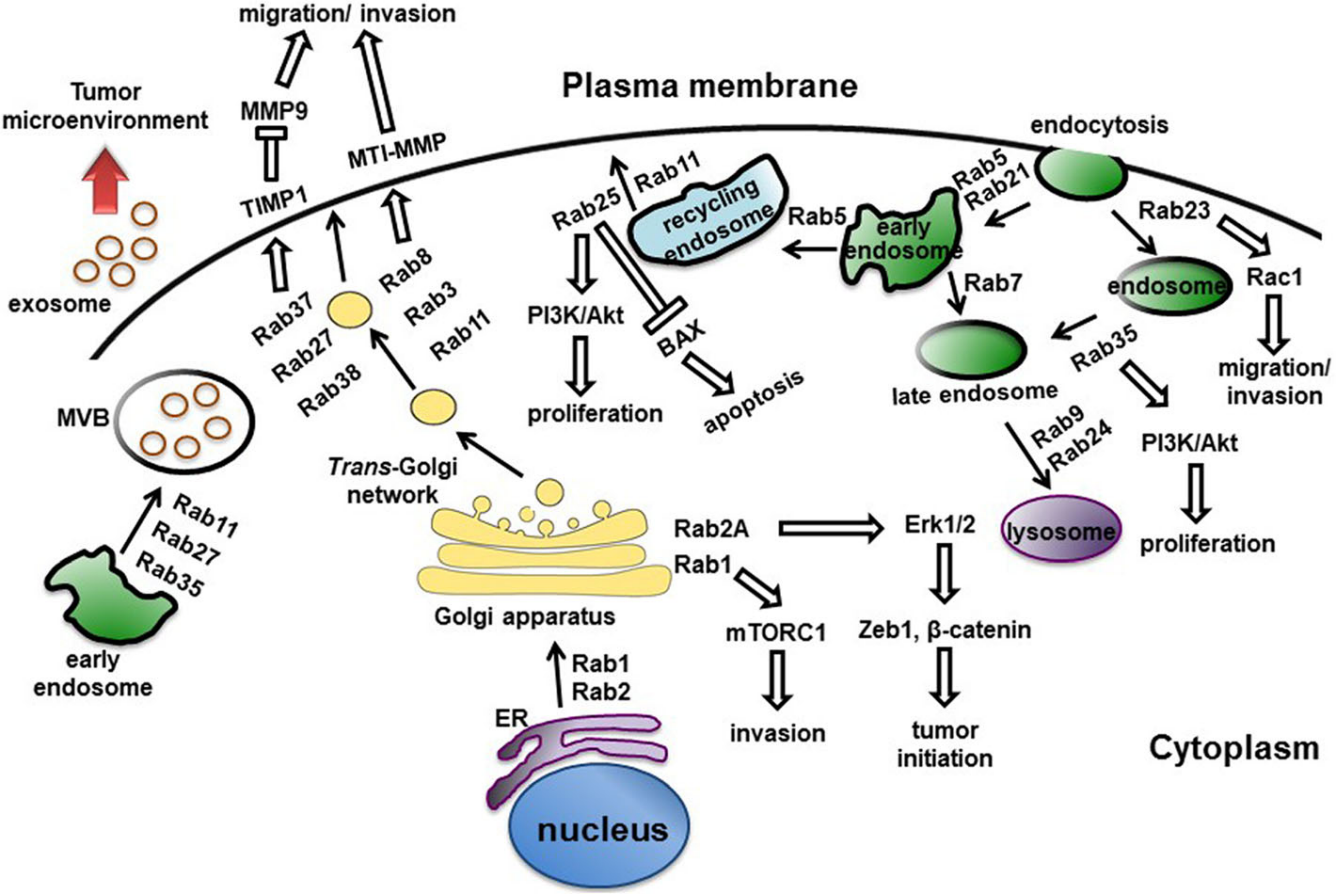
Schematic diagram showing the Rab proteins-mediated vesicular transport and signaling pathways in tumorigenesis. Rab family proteins play key roles in regulating cellular membrane trafficking including endocytosis, exocytosis, exosome secretion as well as vesicles delivery between organelles. All of these vesicles dynamics affects cellular physiology. Dysregulation of oncogenic Rabs at the protein levels or activity such as Rab1, Rab25 and Rab35 exerts tumor-promoting properties such as anti-apoptosis, increase in proliferation, invasion and migration through activation of various signaling pathways. For example, Rab2A facilitates Erk1/2 activation and thus leads to Zeb1 upregulation and β-catenin nuclear translocation, then promotes tumor initiation. In contrast, malfunction of tumor suppressor Rabs promotes oncogenesis and tumor progression. For example, Rab37 delivers its cargo TIMP1 to inhibit MMP9 activity leading to suppression of tumor motility, while loss of Rab37-mediated TIMP1 secretion promotes tumor metastasis. Arrows indicate vesicular movement regulated by Rab proteins. Thick arrows represent Rabs-mediated signaling pathways involved in tumorigenesis and tumor suppression. Note that some organelles and vesicles are relatively enlarged to emphasize the pathways involved

Emerging evidence show that aberrant expression of Rab GTPases is closely associated with tumorigenesis (Fig. 1). Indeed, a set of Rab proteins including Rab1, Rab2A, Rab3D, Rab8, Rab11, Rab21, Rab23, Rab25, Rab27B, Rab35 and others (as reviewed in Table 1) promotes tumor cell migration and invasion to exhibit their effects on tumorigenesis and metastasis by regulating intracellular signal transduction [39–49]. Elevated expression of oncogenic Rab1 has been reported in several cancer types and is associated with poor survival [50–53]. Overexpression of Rab1A promotes mTORC1 signaling and oncogenic growth in response to amino acids stimulation and therefore enhances tumor progression and invasion in colorectal cancer [50, 51]. In addition, gene amplification and overexpression of Rab23 enhance cancer cell invasion and correlate with advanced-stage gastric cancer [47].

**Table 1:** Oncogenic and tumor suppressor Rab proteins in cancers

Rab protein	Cancer types	Expression	Clinical implications	References
Rab1	Colon	Increased	Elevated cell invasion, poor prognosis	[50]
	Liver	Increased	Elevated cell invasion, poor prognosis	[51]
	Brain	Increased	Poor survival	[52]
Rab2	Breast	Increased	Expansion of stem-like cells, poor prognosis	[44]
Rab3	Breast	Increased	Elevated cell motility	[45]
	Brain	Increased	Tumor progression	[81]
Rab4	Breast	Increased	Elevated cell motility	[82]
Rab5	Lung	Increased activity	Elevated cell motility	[83]
Rab11	Breast	Increased activity	Elevated cell invasion	[39]
Rab17	Liver	Decreased	Elevated clinical tumor characteristics	[84]
Rab21	Cervical cancer	Increased	Elevated cell motility	[46]
Rab23	Stomach	Increased	Poor prognosis	[47]
Rab25	Ovarian, Breast	Increased	Poor prognosis	[48]
	Esophagus	Decreased	Poor survival	[55]
	Colon	Decreased	Poor survival	[56]
Rab27	Breast	Increased	Poor prognosis	[42]
Rab31	Breast	Increased	Poor survival	[85]
Rab35	Not applicable	Gain of function mutations	Anti-apoptosis	[49]
Rab37	Lung	Decreased	Poor prognosis	[58, 59]
Rab38	Brain	Increased	Poor prognosis	[86]

Moreover, it has been recently shown that Rabs-mediated vesicle dynamics cooperates with oncogenic signaling pathway to promote tumorigenesis. Indeed, Rab2A drives breast cancer stem cells expansion through activation of Erk signaling [44]. High expression of Rab25 has been frequently associated with poor prognosis in breast and ovarian cancer. Mechanistically, Rab25 expression promotes anti-apoptotic phosphoinositide 3-kinase (PI3K)-Akt pathway and inhibits pro-apoptotic molecules expression such as BAK thereby increasing aggressiveness of cancer cells [48]. Recently, another oncogenic Rab35 has been identified by two gain-of-function mutations in tumor cells. It is proposed that constitutively active Rab35 mediates internalization of platelet-derived growth factor receptor α to LAMP2-positive endosomal membrane, where it drives the activation of oncogenic PI3K/Akt signaling [49], suggesting that Rabs-mediated vesicle dynamics and oncogenic signaling cooperate to direct tumor progression.

Malfunction of Rabs-regulating vesicle trafficking could promote cancer invasion. For example, Rab11 is an important component for membrane proteins recycling and proteins transport from TGN to the plasma membrane [54]. Rab11-mediated α6β4 integrin trafficking has been found to contribute to increase cancer cell invasion in breast cancer [39]. Similarly, Rab25 facilitates invasive cell migration by controlling α5β1 integrin trafficking through the recycling endosomes [40]. Oncogenic Rab8 transports exocytic vesicles carrying membrane type 1-matrix metalloproteinase (MT1-MMP) to the plasma membrane for matrix degradation of migrating cancer cells cultured in collagen gel [41]. In a similar scenario, through mass spectrometric analysis, heat-shock protein 90a has been identified as a component of Rab27B-regulated vesicles, acting as a pro-invasive growth regulator required for activation of matrix metalloproteinase 2 (MMP2). An increased expression of Rab27B is associated with the poor prognosis of oestrogen receptor-positive breast cancer patients, supporting the role of Rab27B in tumor promotion [42].

In contrast to the roles of Rabs in promoting tumor progression, a minor fraction of Rabs is proposed to serve as tumor suppressor (Table 1). However, Rab proteins may have diverse functions in different types or subtypes of cancers. For example, in addition to its role in increasing invasiveness of cancer cells, Rab25 has also been identified to act as a tumor suppressor by inhibiting invasive and angiogenic activities in esophageal squamous cell carcinoma [55] and by increasing malignant tumor formation in the intestines of Rab25^-/-^;Apc^Min/-^ mice [56]. The oncogenic or tumor suppressive functions of Rab25 are cell-type dependent. Rab25 enhances the aggressiveness in ovarian and breast cancer cells [48], while it functions as a tumor suppressor in esophageal squamous cell carcinoma and colorectal carcinoma [55, 56]. An explanation for the discrepancies is that the role of Rab25 in tumorigenesis is dependent on specific or a group of cancer type and on its interplay with cell-type specific effectors.

Rab37 is another example of diverse functions in different types of cancers. Upregulation of Rab37 and its interacting partner TMEM22 are found in renal cell carcinoma (RCC) and decrease in its level by siRNA reduces cancer cell growth, suggesting the oncogenic-like role of Rab37 in RCC [57]. However, promoter hypermethylation of *Rab37* gene leading to low expression of Rab37 mRNA and protein is associated with advanced metastasis in non-small cell lung cancer patients [58]. These results may attributed to Rab-mediated cell-type specific distinct downstream pathways or cargo trafficking. Interestingly, Rab37-mediated exocytosis of tissue inhibitor of metalloproteinase 1 (TIMP1) inactivates extracellular MMP9 and thereby suppresses cell invasion signaling [59]. Of note, reconstituted TIMP1 by addition of TIMP1 recombinant protein abolishes the migration and invasion ability of lung cancer cells in vitro and *in vivo* [59]. In addition, 5-Aza-2-deoxycytidine treatment of a highly metastatic lung cancer cell line shows demethylation and re-expression of the *Rab37* gene and correlates with reduced cancer cell migration [58]. The last-mentioned two studies provide therapeutic strategies such as DNA demethylation of *Rab37* gene and increased expression of Rab37 protein and its cargos such as TIMP1 could facilitate the development of anti-cancer treatment.

### Rab GTPases regulators mediate cancer progression

Dysregulated interaction between Rabs and their effectors could also link to tumor progression and malignancy. Effector proteins including GEFs, GAPs and GDIs together with tethering factors and SNAREs play key roles in regulating Rab GTPase function as molecular switches by cycling between active membrane-bound GTP and inactive cytosolic GDP-bound forms. For example, numerous effectors for Rab5 have been identified, including Rabaptin-5, Vac1, early endosome antigen 1 (EEA1), PI3K, and Class C core vacuole/endosome tethering (CORVET). Increased expression of Rabaptin-5, a Rab effector interacting with GTP-bound Rab5, accelerates endocytosis of epidermal growth factor receptor via Rab5-mediated endosomal fusion pathway and subsequently affects tumor progression [60, 61]. The effector of Rab11, Rab11-family interacting protein 2 (Rab11-FIP2), has been implicated in regulation of recycling endosomal trafficking through interaction with Rab11a [62]. Rab11-FIP2 increases epithelial-mesenchymal transition and metastasis in gastric cancer [63]. Rab11-FIP2 also promotes colorectal cancer cells migration and invasion by upregulating MMP7 expression through activating PI3K/Akt signaling [64]. In contrast, Rab11-FIP1C, another effector of Rab11, acts as a tumor suppressor in ErbB2-mediated breast cancer [65]. DENND2B, a GEF for Rab13, activates Rab13-mediated exocytosis and enhances the invasiveness of cancer cells. Disruption of Rab13-mediated trafficking limits the spread of epithelial cancer cells [66, 67].

Moreover, alteration of SNARE complex has been shown to involve in tumorigenesis. SNARE complex are composed of vesicle associated SNAREs (v-SNARE) and SNAREs at targeting membrane (t-SNAREs). The trans-SNAREs formation by interaction of v-SNAREs with t-SNAREs allows the fusion of vesicle and acceptor membrane. Notably, an increase in interaction between Syntaxin6 and Rab11/vesicle-associated membrane protein 3 (VAMP3) on recycling endosome inhibits αvβ1 and αvβ3 integrins recycling and suppresses cell migration [68]. Conversely, Rab7 and VAMP7 cooperatively mediate endosomal recycling of membrane type MT1-MMP to promote cancer cells migration and invasion [69]. These findings reveal that the effectors and SNAREs of Rabs-mediated membrane trafficking are involved in tumorigenesis. Nevertheless, more research is needed to better understand the complexity of the interaction between Rabs-effectors-SNAREs.

### Rabs and Rab effectors in tumorigenic signalings

Phosphorylation of Rab proteins is important for vesicle targeting and traffic. Rab5a has been reported to be phosphorylated by PKCε to facilitate T-cell migration [70]. Mechanistically, phosphorylated Rab5a promotes Rac1 activation to facilitate actin remodeling. Conventional PKC-mediated Rab11 and Rab6 phosphorylation results in impaired endosomal recycling and redistribution in cytosolic fraction, respectively [71, 72]. Studies have shown that phosphorylation of Rab4 by p34^cdc2^ prevents the association of Rab4 with endosomal membrane by dissociating its binding to membrane effector during the cell cycle [73, 74]. Interestingly, dephosphorylation of Rab7 by PTEN is important for its membrane targeting and subsequent activation, suggesting that phosphorylation status is critical for regulating Rab7 endosomal localization and activity [75]. Rab8A phosphorylation on Ser111 is also observed to impair its binding to Rabin8, the GEF for Rab8A that triggers GDP exchange [76]. However, the significance of post-translational modifications in the regulation of Rab GTPase activity is poorly defined. The clinical relevance of the modifications such as phosphorylation of Rabs in human cancers still remains largely uncharacterized.

In addition, phosphorylation-dependent regulation of Rabs effectors plays important roles in coordinating Rabs-mediated vesicle trafficking. For example, connecdenn1/2 are identified as GEFs for Rab35. Akt-mediated connecdenn 1/2 phosphorylation promotes the interaction of Rab35 and its GEF [77]. Unc-51-like kinases have been reported to phosphorylate DENND3 and upregulate its GEF activity toward Rab12. Activation of Rab12 facilitates autophagosome trafficking in response to starvation [78]. Accordingly, phosphorylation of Rabin8, a GEF for Rab8, by ERK1/2 increases its GEF activity and promotes recycling of transferrin to the plasma membrane [79]. Importantly, dysregulated phosphorylation of Rabs effectors involves in tumorigenesis. For example, Rabaptin-5, a Rab5 effector in endosomal membrane fusion, is a protein kinase D (PKD) substrate. Interestingly, phosphorylated Rabaptin-5 interacts preferentially with Rab4, but not Rab5, to promote αvβ3 recycling leading to enhanced cell motility and invasion [80]. The oncogenic signaling pathways of Rab effectors in promoting tumor development or suppressing tumorigenesis need further elucidation.

## Conclusion

Taken together, as key regulators of cargo transport in vesicle trafficking, it is not surprising that Rab proteins have been linked to tumorigenesis or tumor prevention. Vesicle delivery and dynamics are critical for regulation of cell behaviors associated with cell migration/invasion and tumorigenesis. Notably, specific Rab proteins may have diverse functions in different types or subtypes of cancers. Although mutations or alterations in expression of the components of vesicle transporting machinery may not directly drive cell transformation, cooperation between Rabs and effectors in mediating vesicle movement pathways has critical influences on tumor progression and malignancy. Therefore, it raises the possibility that targeting particular trafficking system may provide a new approach to cancer treatment.

## Abbreviations

ARE: Apical recycling endosome
CORVET: Class C core vacuole/endosome tethering
EEA1: Early endosome antigen 1
GAPs: GTPase-activating proteins
GDIs: Guanine dissociation inhibitors
GEFs: Guanine nucleotide exchange factors
MMP2: Matrix metalloproteinase 2
MT1-MMP: Membrane type 1-matrix metalloproteinase
MVB: Multivesicular body
PI3K: Phosphoinositide 3-kinase
PKD: Protein kinase D
SNARE: Soluble N-ethylmaleimide sensitive factor attachment protein receptor
TGN: Trans-Golgi network
t-SNARE: targeting membrane SNARE
VAMP: Vesicle-associated membrane protein
v-SNARE: vesicle associated SNARE

## References

[1] Stenmark H (2009). Rab GTPases as coordinators of vesicle traffic. Nat Rev Mol Cell Biol.

[2] Chavrier P, Goud B (1999). The role of ARF and Rab GTPases in membrane transport. Curr Opin Cell Biol.

[3] Pereira-Leal JB, Seabra MC (2000). The mammalian Rab family of small GTPases: definition of family and subfamily sequence motifs suggests a mechanism for functional specificity in the Ras superfamily. J Mol Biol.

[4] Hutagalung AH, Novick PJ (2011). Role of Rab GTPases in membrane traffic and cell physiology. Physiol Rev.

[5] Nottingham RM, Pfeffer SR (2009). Defining the boundaries: Rab GEFs and GAPs. Proc Natl Acad Sci U S A.

[6] Seabra MC, Coudrier E (2004). Rab GTPases and myosin motors in organelle motility. Traffic.

[7] Markgraf DF, Peplowska K, Ungermann C (2007). Rab cascades and tethering factors in the endomembrane system. FEBS Lett.

[8] Ohya T, Miaczynska M, Coskun U, Lommer B, Runge A, Drechsel D, Kalaidzidis Y, Zerial M (2009). Reconstitution of Rab- and SNARE-dependent membrane fusion by synthetic endosomes. Nature.

[9] Lütcke A, Jansson S, Parton RG, Chavrier P, Valencia A, Huber LA, Lehtonen E, Zerial M (1993). Rab17, a novel small GTPase is specific for epithelial cells and is induced during cell polarization. J Cell Biol.

[10] Zuk PA, Elferink LA (1999). Rab15 mediates an early endocytic event in Chinese hamster ovary cells. J Biol Chem.

[11] Casanova JE, Wang X, Kumar R, Bhartur SG, Navarre J, Woodrum JE, Altschuler Y, Ray GS, Goldenring JR (1999). Association of Rab25 and Rab11a with the apical recycling system of polarized Madin–Darby canine kidney cells. Mol Biol Cell.

[12] Iida H, Noda M, Kaneko T, Doiguchi M, Mori T (2005). Identification of rab12 as a vesicle-associated small GTPase highly expressed in Sertoli cells of rat testis. Mol Reprod Dev.

[13] Chen Y, Wang Y, Zhang J, Deng Y, Jiang L, Song E, Wu XS, Hammer JA, Xu T, Lippincott-Schwartz J (2012). Rab10 and myosin-Va mediate insulin-stimulated GLUT4 storage vesicle translocation in adipocytes. J Cell Biol.

[14] Sun Y, Bilan PJ, Liu Z, Klip A (2010). Rab8A and Rab13 are activated by insulin and regulate GLUT4 translocation in muscle cells. Proc Natl Acad Sci U S A.

[15] Zheng JY, Koda T, Fujiwara T, Kishi M, Ikehara Y, Mitsuaki K (1998). A novel Rab GTPase, Rab33B, is ubiquitously expressed and localized to the medial Golgi cisternae. J Cell Sci.

[16] Zeigerer A, Bogorad RL, Sharma K, Gilleron J, Seifert S, Sales S, Berndt N, Bulik S, Marsico G, D’Souza RC, Lakshmanaperumal N, Meganathan K, Natarajan K, Sachinidis A, Dahl A, Holzhütter HG, Shevchenko A, Mann M, Koteliansky V, Zerial M (2015). Regulation of liver metabolism by the endosomal GTPase Rab5. Cell Rep.

[17] Pellinen T, Arjonen A, Vuoriluoto K, Kallio K, Fransen J, Ivaska J (2006). Small GTPase Rab21 regulates cell adhesion and controls endosomal traffic of beta1-integrins. J Cell Biol.

[18] Feng Y, Press B, Wandinger-Ness A (1995). Rab 7: an important regulator of late endocytic membrane traffic. J Cell Biol.

[19] Rupnik M, Kreft M, Nothias F, Grilc S, Bobanovic LK, Johannes L, Kiauta T, Vernier P, Darchen F, Zorec R (2007). Distinct role of Rab3A and Rab3B in secretory activity of rat melanotrophs. Am J Physiol Cell Physiol.

[20] Takahashi S, Kubo K, Waguri S, Yabashi A, Shin HW, Katoh Y, Nakayama K (2012). Rab11 regulates exocytosis of recycling vesicles at the plasma membrane. J Cell Sci.

[21] Nashida T, Imai A, Shimomura H (2006). Relation of Rab26 to the amylase release from rat parotid acinar cells. Arch Oral Biol.

[22] Tolmachova T, Abrink M, Futter CE, Authi KS, Seabra MC (2007). Rab27b regulates number and secretion of platelet dense granules. Proc Natl Acad Sci U S A.

[23] Masuda ES, Luo Y, Young C, Shen M, Rossi AB, Huang BC, Yu S, Bennett MK, Payan DG, Scheller RH (2000). Rab37 is a novel mast cell specific GTPase localized to secretory granules. FEBS Lett.

[24] Wasmeier C, Romao M, Plowright L, Bennett DC, Raposo G, Seabra MC (2006). Rab38 and Rab32 control post-Golgi trafficking of melanogenic enzymes. J Cell Biol.

[25] Kauppi M, Simonsen A, Bremnes B, Vieira A, Callaghan J, Stenmark H, Olkkonen VM (2002). The small GTPase Rab22 interacts with EEA1 and controls endosomal membrane trafficking. J Cell Sci.

[26] Rodriguez-Gabin AG, Ortiz E, Demoliner K, Si Q, Almazan G, Larocca JN (2010). Interaction of Rab31 and OCRL-1 in oligodendrocytes: its role in transport of mannose 6-phosphate receptors. J Neurosci Res.

[27] de Armentia MM L, Amaya C, Colombo M (2016). Rab GTPases and the autophagy pathway: Bacterial targets for a suitable biogenesis and trafficking of their own vacuoles. Cells.

[28] Dou Z, Pan JA, Dbouk HA, Ballou LM, DeLeon JL, Fan Y, Chen JS, Liang Z, Li G, Backer JM, Lin RZ, Zong WX (2013). Class IA PI3K p110b subunit promotes autophagy through Rab5 small GTPase in response to growth factor limitation. Mol Cell.

[29] Hirota Y, Tanaka Y (2009). A small GTPase, human Rab32, is required for the formation of autophagic vacuoles under basal conditions. Cell Mol Life Sci.

[30] Fukuda M, Itoh T (2008). Direct link between Atg protein and small GTPase Rab: Atg16L functions as a potential Rab33 effector in mammals. Autophagy.

[31] Hyttinen JM, Niittykoski M, Salminen A, Kaarniranta K (2013). Maturation of autophagosomes and endosomes: a key role for Rab7. Biochim. Biophys. Acta – Mol. Cell Res.

[32] Longatti A, Lamb CA, Razi M, Yoshimura SI, Barr FA, Tooze SA (2012). TBC1D14 regulates autophagosome formation via Rab11- and ULK1- positive recycling endosomes. J Cell Biol.

[33] Munafó DB, Colombo MI (2002). Induction of autophagy causes dramatic changes in the subcellular distribution of GFP-Rab24. Traffic.

[34] Liu Y, Tao X, Jia L, Cheng KW, Lu Y, Yu Y, Feng Y (2012). Knockdown of RAB25 promotes autophagy and inhibits cell growth in ovarian cancer cells. Mol Med Rep.

[35] Zhang X, Yuan X, Shi H, Wu L, Qian H, Xu W (2015). Exosomes in cancer: small particle, big player. J Hematol Oncol.

[36] Savina A, Vidal M, Colombo MI (2002). The exosome pathway in K562 cells is regulated by Rab11. J Cell Sci.

[37] Hsu C, Morohashi Y, Yoshimura S, Manrique-Hoyos N, Jung S, Lauterbach MA, Bakhti M, Grønborg M, Mobius W, Rhee J, Barr FA, Simons M (2010). Regulation of exosome secretion by Rab35 and its GTPase-activating proteins TBC1D10A-C. J Cell Biol.

[38] Ostenfeld MS, Jeppesen DK, Laurberg JR, Boysen AT, Bramsen JB, Primdal-Bengtson B, Hendrix A, Lamy P, Dagnaes-Hansen F, Rasmussen MH, Bui KH, Fristrup N, Christensen EI, Nordentoft I, Morth JP, Jensen JB, Pedersen JS, Beck M, Theodorescu D, Borre M, Howard KA, Dyrskjøt L, Ørntoft TF (2014). Cellular disposal of miR23b by RAB27-dependent exosomes release is linked to acquisition of metastatic properties. Cancer Res.

[39] Yoon SO, Shin S, Mercurio AM (2005). Hypoxia stimulates carcinoma invasion by stabilizing microtubules and promoting the Rab11 trafficking of the alpha6beta4 integrin. Cancer Res.

[40] Caswell PT, Spence HJ, Parsons M, White DP, Clark K, Cheng KW, Mills GB, Humphries MJ, Messent AJ, Anderson KI, McCaffrey MW, Ozanne BW, Norman JC (2007). Rab25 associates with alpha5beta1 integrin to promote invasive migration in 3D microenvironments. Dev Cell.

[41] Bravo-Cordero JJ, Marrero-Diaz R, Megías D, Genís L, García-Grande A, García MA (2007). MT1-MMP proinvasive activity is regulated by a novel Rab8-dependent exocytic pathway. EMBO J.

[42] Hendrix A, Maynard D, Pauwels P, Braems G, Denys H, Van den Broecke R, Lambert J, Van Belle S, Cocquyt V, Gespach C, Bracke M, Seabra MC, Gahl WA, De Wever O, Westbroek W (2010). Effect of the secretory small GTPase Rab27B on breast cancer growth, invasion, and metastasis. J Natl Cancer Inst.

[43] Yang XZ, Li XX, Zhang YJ, Rodriguez-Rodriguez L, Xiang MQ, Wang HY, Zheng XF (2016). Rab1 in cell signaling, cancer and other diseases. Oncogene.

[44] Luo ML, Gong C, Chen CH, Hu H, Huang P, Zheng M, Yao Y, Wei S, Wulf G, Lieberman J, Zhou XZ, Song E, Lu KP (2015). The Rab2A GTPase promotes breast cancer stem cells and tumorigenesis via Erk signaling activation. Cell Rep.

[45] Yang J, Liu W, Lu X, Fu Y, Li L, Luo Y (2015). High expression of small GTPase Rab3D promotes cancer progression and metastasis. Oncotarget.

[46] Tang BL, Ng EL. Rabs and cancer cell motility. Cell Motil Cytoskeleton. 2009;66(7):365–70. 10.1002/cm.20376 19418559

[47] Hou Q, Wu YH, Grabsch H, Zhu Y, Leong SH, Ganesan K, Cross D, Tan LK, Tao J, Gopalakrishnan V, Tang BL, Kon OL, Tan P (2008). Integrative genomics identifies RAB23 as an invasion mediator gene in diffuse-type gastric cancer. Cancer Res.

[48] Cheng KW, Lahad JP, Kuo WL, Lapuk A, Yamada K, Auersperg N, Liu J, Smith-McCune K, Lu KH, Fishman D, Gray JW, Mills GB (2004). The RAB25 small GTPase determines aggressiveness of ovarian and breast cancers. Nat Med.

[49] Wheeler DB, Zoncu R, Root DE, Sabatini DM, Sawyers CL (2015). Identification of an oncogenic RAB protein. Science.

[50] Thomas JD, Zhang YJ, Wei YH, Cho JH, Morris LE, Wang HY, Zheng XF (2014). Rab1A is an mTORC1 activator and a colorectal oncogene. Cancer Cell.

[51] Xu BH, Li XX, Yang Y, Zhang MY, Rao HL, Wang HY, Zheng XF (2015). Aberrant amino acid signaling promotes growth and metastasis of hepatocellular carcinomas through Rab1A-dependent activation of mTORC1 by Rab1A. Oncotarget.

[52] Bao ZS, Li MY, Wang JY, Zhang CB, Wang HJ, Yan W, Liu YW, Zhang W, Chen L, Jiang T (2014). Prognostic value of a nine-gene signature in glioma patients based on mRNA expression profiling. CNS Neurosci Ther.

[53] Abd Elmageed ZY, Yang Y, Thomas R, Ranjan M, Mondal D, Moroz K, Fang Z, Rezk BM, Moparty K, Sikka SC, Sartor O, Abdel-Mageed AB (2014). Neoplastic reprogramming of patient-derived adipose stem cells by prostate cancer cell-associated exosomes. Stem Cells.

[54] Bhuin T, Roy JK (2014). Rab proteins: the key regulators of intracellular vesicle transport. Exp Cell Res.

[55] Tong M, Chan KW, Bao JY, Wong KY, Chen JN, Kwan PS, Tang KH, Fu L, Qin YR, Lok S, Guan XY, Ma S (2012). Rab25 is a tumor suppressor gene with antiangiogenic and anti-invasive activities in esophageal squamous cell carcinoma. Cancer Res.

[56] Nam KT, Lee HJ, Smith JJ, Lapierre LA, Kamath VP, Chen X, Aronow BJ, Yeatman TJ, Bhartur SG, Calhoun BC, Condie B, Manley NR, Beauchamp RD, Coffey RJ, Goldenring JR (2010). Loss of Rab25 promotes the development of intestinal neoplasia in mice and is associated with human colorectal adenocarcinomas. J Clin Invest.

[57] Dobashi S, Katagiri T, Hirota E, Ashida S, Daigo Y, Shuin T, Fujioka T, Miki T, Nakamura Y (2009). Involvement of TMEM22 overexpression in the growth of renal cell carcinoma cells. Oncol Rep.

[58] Wu CY, Tseng RC, Hsu HS, Wang YC, Hsu MT (2009). Frequent down-regulation of hRAB37 in metastatic tumor by genetic and epigenetic mechanisms in lung cancer. Lung Cancer.

[59] Tsai CH, Cheng HC, Wang YS, Lin P, Jen J, Kuo IY, Chang YH, Liao PC, Chen RH, Yuan WC, Hsu HS, Yang MH, Hsu MT, Wu CY, Wang YC (2014). Small GTPase Rab37 targets tissue inhibitor of metalloproteinase 1 for exocytosis and thus suppresses tumour metastasis. Nat Commun.

[60] Park MH, Choi KY, Mindo S (2015). The pleckstrin homology domain of phospholipase D1 accelerates EGFR endocytosis by increasing the expression of the Rab5 effector, rabaptin-5. Exp Mol Med.

[61] Park SJ, Kim JK, Bae HJ, Eun JW, Shen Q, Kim HS, Shin WC, Yang HD, Lee EK, You JS, Park WS, Lee JY, Nam SW (2014). HDAC6 sustains growth stimulation by prolonging the activation of EGF receptor through the inhibition of rabaptin-5-mediated early endosome fusion in gastric cancer. Cancer Lett.

[62] Ducharme NA, Hales CM, Lapierre LA, Ham AJ, Oztan A, Apodaca G, Goldenring JR (2006). MARK2/EMK1/Par-1Balpha phosphorylation of Rab11-family interacting protein 2 is necessary for the timely establishment of polarity in Madin-Darby canine kidney cells. Mol Biol Cell.

[63] Dong W, Qin G, Shen R (2016). Rab11-FIP2 promotes the metastasis of gastric cancer cells. Int J Cancer.

[64] Xu CL, Wang JZ, Xia XP, Pan CW, Shao XX, Xia SL, Yang SX, Zheng B (2016). Rab11-FIP2 promotes colorectal cancer migration and invasion by regulating PI3K/AKT/MMP7 signaling pathway. Biochem Biophys Res Commun.

[65] Boulay PL, Mitchell L, Turpin J, Huot-Marchand JÉ, Lavoie C, Sanguin-Gendreau V, Jones L, Mitra S, Livingstone JM, Campbell S, Hallett M, Mills GB, Park M, Chodosh L, Strathdee D, Norman JC, Muller WJ (2016). Rab11-FIP1C is a critical negative regulator in ErbB2-mediated mammary tumor progression. Cancer Res.

[66] Ioannou MS, Bell ES, Girard M, Chaineau M, Hamlin JN, Daubaras M, Monast A, Park M, Hodgson L, McPherson PS (2015). DENND2B activates Rab13 at the leading edge of migrating cells and promotes metastatic behavior. J Cell Biol.

[67] Ioannou MS, McPherson PS (2016). Regulation of cancer cell behavior by the small GTPase Rab13. J Biol Chem.

[68] Reverter M, Rentero C, Garcia-Melero A, Hoque M (2014). Vilà de Muga S, Alvarez-Guaita A, Conway JR, Wood P, Cairns R, Lykopoulou L, Grinberg D, Vilageliu L, Bosch M, Heeren J, Blasi J, Timpson P, Pol A, Tebar F, Murray RZ, Grewal T, Enrich C. Cholesterol regulates Syntaxin 6 trafficking at trans-Golgi network endosomal boundaries. Cell Rep.

[69] Williams KC, Coppolino MG (2011). Phosphorylation of membrane type 1-matrix metalloproteinase (MT1-MMP) and its vesicle-associated membrane protein 7 (VAMP7)-dependent trafficking facilitate cell invasion and migration. J Biol Chem.

[70] Ong ST, Freeley M, Skubis-Zegadło J, Fazil MH, Kelleher D, Fresser F, Baier G, Verma NK, Long A (2014). Phosphorylation of Rab5a protein by protein kinase Cε is crucial for T-cell migration. J Biol Chem.

[71] Pavarotti M, Capmany A, Vitale N, Colombo MI, Damiani MT (2012). Rab11 is phosphorylated by classical and novel protein kinase C isoenzymes upon sustained phorbol ester activation. Biol Cell.

[72] Fitzgerald ML, Reed GL (1999). Rab6 is phosphorylated in thrombin-activated platelets by a protein kinase C-dependent mechanism: effects on GTP/GDP binding and cellular distribution. Biochem J.

[73] Ayad N, Hull M, Mellman I (1997). Mitotic phosphorylation of rab4 prevents binding to a specific receptor on endosome membranes. EMBO J.

[74] van der Sluijs P, Hull M, Huber LA, Mâle P, Goud B, Mellman I (1992). Reversible phosphorylation--dephosphorylation determines the localization of rab4 during the cell cycle. EMBO J.

[75] Shinde SR, Maddika S (2016). A modification switch on a molecular switch: Phosphoregulation of Rab7 during endosome maturation. Small GTPases.

[76] Lai YC, Kondapalli C, Lehneck R, Procter JB, Dill BD, Woodroof HI, Gourlay R, Peggie M, Macartney TJ, Corti O, Corvol JC, Campbell DG, Itzen A, Trost M, Muqit MM (2015). Phosphoproteomic screening identifies Rab GTPases as novel downstream targets of PINK1. EMBO J.

[77] Kulasekaran G, Nossova N, Marat AL, Lund I, Cremer C, Ioannou MS, McPherson PS (2015). Phosphorylation-dependent Regulation of Connecdenn/DENND1 Guanine Nucleotide Exchange Factors. J Biol Chem.

[78] Xu J, Fotouhi M, McPherson PS (2015). Phosphorylation of the exchange factor DENND3 by ULK in response to starvation activates Rab12 and induces autophagy. EMBO Rep.

[79] Wang J, Ren J, Wu B, Feng S, Cai G, Tuluc F, Peränen J, Guo W (2015). Activation of Rab8 guanine nucleotide exchange factor Rabin8 by ERK1/2 in response to EGF signaling. Proc Natl Acad Sci U S A.

[80] Christoforides C, Rainero E, Brown KK, Norman JC, Toker A (2012). PKD controls αvβ3 integrin recycling and tumor cell invasive migration through its substrate Rabaptin-5. Dev Cell.

[81] Kim JK, Lee SY, Park CW, Park SH, Yin J, Kim J, Park JB, Lee JY, Kim H, Kim SC (2014). Rab3a promotes brain tumor initiation and progression. Mol Biol Rep.

[82] Frittoli E, Palamidessi A, Marighetti P, Confalonieri S, Bianchi F, Malinverno C, Mazzarol G, Viale G, Martin-Padura I, Garré M, Parazzoli D, Mattei V, Cortellino S, Bertalot G, Di Fiore PP, Scita G (2014). A RAB5/RAB4 recycling circuitry induces a proteolytic invasive program and promotes tumor dissemination. J Cell Biol.

[83] Silva P, Mendoza P, Rivas S, Díaz J, Moraga C, Quest AF, Torres VA (2016). Hypoxia promotes Rab5 activation, leading to tumor cell migration, invasion and metastasis. Oncotarget.

[84] Wang K, Mao Z, Liu L, Zhang R, Liang Q, Xiong Y, Yuan W, Wei L (2015). Rab17 inhibits the tumourigenic properties of hepatocellular carcinomas via the Erk pathway. Tumour Biol.

[85] Kotzsch M, Dorn J, Doetzer K, Schmalfeldt B, Krol J, Baretton G, Kiechle M, Schmitt M, Magdolen V (2011). mRNA expression levels of the biological factors uPAR, uPAR-del4/5, and rab31, displaying prognostic value in breast cancer, are not clinically relevant in advanced ovarian cancer. Biol Chem.

[86] Wang H, Jiang C (2013). RAB38 confers a poor prognosis, associated with malignant progression and subtype preference in glioma. Oncol Rep.

